# Interaction Between Malat1 and miR-499-5p Regulates Meis1 Expression and Function with a Net Impact on Cell Proliferation

**DOI:** 10.3390/cells14020125

**Published:** 2025-01-16

**Authors:** Salma A. Fahim, Manon Ragheb, Ibrahim Hassan Fayed, Aya Osama, Ahmed Karam, Sameh Magdeldin, Rana Metwale, Mohamed Dief Allah Abdalmoneam Elsayed, Ahmed Abdellatif, Hesham A. Sadek, Shereen Ahmed El Sobky, Nada El-Ekiaby, Injie Omar Fawzy, Ahmed Ihab Abdelaziz

**Affiliations:** 1 School of Medicine, Newgiza University (NGU), Giza 12577, Egypt; 2 Biotechnology Program, American University in Cairo, Cairo 11835, Egypt; 3 Proteomics and Metabolomics Unit, Basic Research Department, Children’s Cancer Hospital 57357 Cairo, (CCHE-57357), Cairo 11562, Egypt; 4 Department of Physiology, Faculty of Veterinary Medicine, Suez Canal University, Ismailia 41522, Egypt; 5 Department of Anatomy and Embryology, Faculty of Medicine, Cairo University, Cairo 11562, Egypt; 6 Division of Cardiology, University of Arizona College of Medicine, Tucson, AR 85721, USA; 7 Division of Cardiology, Department of Internal Medicine, The University of Texas Southwestern Medical Center, Dallas, TX 85004, USA

**Keywords:** Meis1, Malat1, miR-499-5p, cell proliferation, cell cycle, non-coding RNAs, gene regulation

## Abstract

Meis1 is a transcription factor involved in numerous functions including development and proliferation and has been previously shown to harness cell cycle progression. In this study, we used in silico analysis to predict that miR-499-5p targets Meis1 and that Malat1 sponges miR-499-5p. For the first time, we demonstrated that the overexpression of miR-499-5p led to the downregulation of Meis1 mRNA and protein in C166 cells by directly binding to its 3’UTR. Moreover, knocking down Malat1 increased miR-499-5p expression, subsequently suppressing Meis1. Through BrdU incorporation assay, we showed that the knockdown of Malat1, Meis1, or mimicking with miR-499-5p promoted cell proliferation. Enrichment analyses on proteins identified via mass spectrometry after manipulating Malat1, miR-499-5p, or Meis1 revealed a multitude of differentially expressed proteins related to cell cycle, cell division, and key pathways like Wnt and mTOR, essential for cell proliferation. Collectively, our findings confirm that Malat1 sponges miR-499-5p, regulating Meis1, and that Malat1/miR-499-5p/Meis1 could potentially form an axis that has a pivotal influence on cellular proliferation.

## 1. Introduction

Myeloid ecotropic virus insertion site 1 (Meis1) is a member of the triple amino acid loop extension (TALE) homeobox family. It was initially discovered in the late 1990s as a factor contributing to leukemogenesis in the BXH-2 mouse model. Meis1 is highly conserved among mammals [1] and has numerous interacting partners that often orchestrate its spatial–temporal transcriptional activity, defining its diverse functions, and ultimately contributing to its inherent complexity. Meis1 is a transcription factor involved in critical physiological and pathological processes including proliferation, differentiation, cell cycle regulation, and limb and organ patterning [2,3,4]. It has been implicated in the pathogenesis of different types of cancers, such as neuroblastomas [5], prostate cancer [6], ovarian cancer [7], non-small cell lung adenocarcinoma [8], and leukemia [1,9], often affecting cellular proliferation. Interestingly, several lines of evidence have shed light on its paradoxical roles in tumorigenesis, describing it as both oncogenic [10,11,12] and a tumor suppressor [8,13,14], suggesting that it can either stimulate or inhibit cell proliferation depending on the cell type, tissue, and/or the interactive network.

Notably, Meis1 has been shown to suppress the proliferation of mammalian cardiomyocytes by regulating cyclin-dependent kinases inhibitors (CDKIs) and inducing cell cycle arrest [15]. In contrast, inhibiting Meis1 has been demonstrated to extend cardiomyocyte proliferation, offering a promising strategy for enhancing heart repair and regeneration following ischemic injury [16]. It is therefore evident that Meis1 is part of a sophisticated network and controlling its expression could ultimately affect cellular proliferation or senescence, and even the pathogenesis of certain diseases like cancer and ischemic cardiomyopathy.

Non-coding RNAs (ncRNAs) play integral roles in the regulation of gene expression, both transcriptionally and post-transcriptionally. MicroRNAs (miRNAs or miRs) are short RNA sequences ranging from 18 to 25 nucleotides that typically bind to a target mRNA at the 3′ untranslated region (UTR) and downregulate its expression [17]. Conversely, long non-coding RNAs (lncRNAs) are over 200 nucleotides in size and have the ability to interact with DNA, RNA, and proteins. They are also known to act as competing endogenous RNAs (ceRNA) by sponging miRNAs, thereby preventing the inhibitory effect of miRNAs on their targets [18]. miRNAs and lncRNAs are essential for maintaining homeostasis and ensuring proper cellular activities by interacting with various molecular targets and pathways. They exhibit remarkable versatility in their regulatory functions, influencing a wide range of biological processes such as differentiation [19,20], development [18,21,22], cell cycle and division [23,24], and, of particular relevance to our study, proliferation [25,26,27]. As a result, manipulations of these ncRNAs have been carried out in many biological systems to restore specific cellular functions or overcome genetic aberrations.

Interestingly, the repertoire of Meis1 regulators includes several reported miRNAs such as miR-155 [28,29], miR-196b [30] and miR-144 [31], which were shown to downregulate Meis1 and impact hematopoiesis and cancer progression. Additionally, miR-548c-3p, miR-509-3p, and miR-23b-3p in adult rat cardiomyocytes directly targeted Meis1 and significantly induced the proliferation of otherwise senescent cells [32]. Moreover, the lncRNA ELFN1-AS1 is the only lncRNA known to act upstream of Meis1, affecting its expression and function through the EZH2/DNMT3A axis, thereby promoting tumorigenesis [33]. Therefore, the identification of new ncRNA regulators of Meis1 is important for deepening our understanding of the complex mechanisms governing its regulation. Such findings would not only shed light on the intricate control of Meis1 but could also pave the way for therapeutic interventions by influencing its diverse roles in development, tissue patterning, and cell cycle progression. This could have broad applications in treating heart diseases, cancer, and developmental disorders.

In the present study, our aim was to characterize novel ncRNAs as upstream regulators of Meis1 in an effort to modulate the proliferative capacity of cells. This was achieved by in silico target predictions which were validated in vitro via luciferase assay and transfection experiments. Finally, the functional impact of the ncRNAs was assessed by proliferation assays and proteomics analyses.

## 2. Materials and Methods

### 2.1. Cell Culture and Oligonucleotide Transfection

C166 endothelial mouse cell lines and HEK293 human embryonic kidney cell lines were obtained from ATCC and cultured in DMEM high glucose (glucose concentration: 4.5 g/L) (Biowest, Nuaillé, France) supplemented with 10% fetal bovine serum (FBS) and 1% penicillin/streptomycin (Lonza, Visp, Switzerland). Cells were maintained at 37 °C and 5% CO2. C166 cells were transfected with 5 nM of miR-499-5p mimics (MSY0002870, Eurofins, Luxembourg, Luxembourg) or specific siRNAs against Meis1 (si-Meis1, SI01303603, SI01303610) and Malat1 (si-Malat1, SI03977267) (Qiagen, Hilden, Germany) with a final concentration of 40 nM and 60 nM, respectively, using Lipofectamine 3000 Transfection Reagent (Invitrogen, Carlsbad, CA, USA) according to the manufacturer’s protocol. Non-mammalian oligonucleotides (1027281, Qiagen) were transfected to serve as negative controls (NCs).

### 2.2. RNA Extraction and Quantification

Total RNA was extracted from C166 cells using the Trizol extraction reagent (Invitrogen, USA), following the manufacturer’s instructions. Extraction was performed 48 h after transfection of miR-499-5p mimic and si-Meis1, and 72 h following si-Malat1 transfection. Concentration and integrity of the extracted RNA were measured using the Nanodrop (ThermoFischer, Waltham, MA, USA).

### 2.3. Reverse Transcription and qPCR

RNA was reverse-transcribed into single-stranded complementary DNA (cDNA) using the miScript II RT kit (Qiagen, Germany) or the high-capacity cDNA reverse transcription kit (Applied Biosystems, Carlsbad, CA, USA), according to the manufacturer’s protocol. Relative expression of miR-499-5p was normalized to RNU6B in each sample, whereas Meis1 and Malat1 relative expressions were normalized to the SRSF4 housekeeping gene. Real-time quantitative polymerase chain reaction (qPCR) was performed on the cDNA from C166 cells using Quantinova (Qiagen, Germany) master mixes, respectively, and conducted on StepOne Real-Time PCR instrument (Applied Biosystems, USA). Relative expression was calculated using the 2^−ΔΔCT^ method. Sequences of the used RTqPCT primers are provided in Supplementary Table S1.

### 2.4. Shotgun Proteomics Analysis

Proteins were precipitated following Meis1 or Malat1 knockdown or miR-499-5p mimicking in C166 cells with four-time-chilled acetone. After incubation at −80 °C for 30 min and at −20 °C overnight, samples were centrifuged and the protein extracts were denatured using a lysis solution (8 M urea, 500 mM Tris HCl, pH 8.5) with complete ultra-proteases (Roche, Mannheim, Germany). The protein concentration was assayed using the bicinchoninic acid (BCA) method (Pierce, Rockford, IL, USA) at Å562 nm before digestion. Next, samples were subjected to digestion where the protein pellets were reduced with 200 mM 1,4-Dithiothreitol (DTT) for 30 min. Then, alkylation of cysteine residues was performed using 10 mM iodoacetamide for 30 min in a dark area. Samples were diluted to a final concentration of 2 M urea with 100 mM Tris-HCl, pH 8.5, prior to digestion with trypsin. For endopeptidase digestion, MS-grade modified porcine trypsin (Sigma, Steinheim, Germany) was added with a protein-to-protease mass ratio of 30:1, and incubated overnight in a thermo-shaker at 600 rpm at 37 °C. Digested peptide solution was acidified using formic acid to a final pH of 2.0. The resultant peptide mixture herein was cleaned up using a stage tip, as discussed earlier [34]. Peptides were assayed using the BCA method (Pierce, Rockford IL, USA) at Å562 nm prior to be injected with a final concentration of 100 ng/µL.

### 2.5. Nano LC-MS/MS Analysis

Samples were analyzed by LC−MS-MS using an EASY-nanoLC 1200 system and an Orbitrap Fusion Lumos Tribrid mass spectrometer (Thermo Fisher Scientific, Waltham, MA, USA) coupled with a NanoFlex ion source. In total, 2 µL/100ng peptides were trapped on Thermo Scientific™ Acclaim™ PepMap™ 100 (75 µm i.d, 2 cm long, 3 µm particles, C18) trap column and eluted on Thermo Scientific™ Acclaim™ PepMap™ 100 analytical column with 75 µm i.d., 2 µm particles, which were 25 cm long, and C18 packing. Columns were connected to the LC system using a Thermo Scientific™ nanoViper™ fingertight fitting. Sample elution was performed using a 60 min gradient of solvent B (0.1% formic acid, 80% acetonitrile) running for 5−30% (0–31 min), 30–40% (31–41 min), 40–80% (41–51 min) and held for 4 min at a flow rate of 250 nL/min, followed by a 5 min ramp to 100%. Solution A consisted of 0.1% formic acid in water.

The mass spectrometer operated in data-dependent acquisition (DDA) mode with 3 s cycles for the survey and the MS/MS scans. Survey scans of peptide precursors were performed from 400 to 1800 m/z at 120 K resolution with standard automatic gain control (AGC) and maximum ion injection time (IT) set to auto-mode. Monoisotopic precursor selection (MIPS) was determined at the peptide level with an intensity threshold of 5 × 10^3^, and only peptides with charge states of 2 to 7 were accepted as tandem MS. The dynamic exclusion was set to 30 s with a 10 ppm mass tolerance, and isotopes were excluded. Isolation for MS2 scans was performed in the quadrupole with an isolation window of 1.5 m/z. Higher-energy collisional dissociation (HCD) activation was applied with 30% collision energy using dynamic injection time mode and standard AGC target. The resulting fragments were detected using the rapid scan rate in the linear ion trap. MS1 and MS2 spectra were recorded in profile and centroid modes, respectively. The MS proteomics data were deposited to the ProteomeXchange Consortium through the PRIDE repository with identifier PXD054558.

### 2.6. Proteomics Data Analysis

Proteome discoverer 1.4 3 (version 2.4.0.305, Thermo Scientific) was used as a raw data post-processing interface to select scan events for peptide/protein identification. Peak lists were searched against the human UniProt FASTA database version of 2022 (225,732 entries) using Sequest HT search engine. The search of all fully and semi-tryptic peptide candidates was adjusted up to 2 missed cleavages with at least 6 amino acids. Precursor mass and fragment mass were identified with an initial mass tolerance of 20 ppm and 0.5 Da, respectively. Carbamidomethylation of cysteine (+57.02146 amu) was considered as a static modification and oxidation at methionine (+15.995), acetylation of protein N- terminal and K (+42.01 amu), and pyrrolidone from carbamidomethylated C (−17.03 amu) as variable modifications. The false discovery rate (FDR) was set to 1% for peptides (minimum length of 7 amino acids) and proteins and was determined by searching a reverse database to ensure high-quality results. Protein abundances were calculated using the summed abundance parameter. Unique and razor peptides were used for quantification, and the precursor abundance was calculated based on intensity. Normalization was carried out on the total peptide amount. Protein ratio calculation was carried out using a pairwise ratio based on a t-test (background-based) as a hypothesis test. Moreover, Igraph v1.6.0 and Cytoscape v3.10.3 were used to visualize the connections between proteins and cell cycle-related gene ontology (GO) aspects.

### 2.7. Western Blotting

Cells were lysed 48 h post-transfection in RIPA lysis buffer (Serva, Heidelberg, Germany) mixed with 1X protease inhibitor and 1X phosphatase inhibitor (Thermo Scientific, USA). Protein concentrations were measured using Modified Lowry protein assay kit (Thermo Scientific, USA), according to the manufacturer’s instructions. Equal amounts of protein lysates were loaded and run on a 10% SDS-polyacrylamide gel using Bio-Rad Western blot system (Bio-Rad, Hercules, CA, USA). After transferring the proteins to a nitrocellulose membrane (Serva, Heidelberg, Germany), the membrane was blocked with 5% non-fat dry milk in 1X TBS-T and incubated overnight at 4 °C with the appropriate primary antibodies, which are as follows: rabbit-anti-Meis1 (Cell Signaling, Danvers, MA, USA, 12744, 1:300) and mouse-anti-Vinculin (Sigma, V9131, 1:1000). Horseradish peroxidase (HRP)-conjugated anti-rabbit (Cell Signaling, 7074, 1:5000) or anti-mouse (Santa Cruz, sc-516102, 1:5000) secondary antibodies for the detection of Meis1 and Vinculin, respectively, were used. Protein bands were visualized with Pierce ECL Western blotting substrate (Thermo Scientific, 32209), and processed and quantified by ImageQuant TL software (latest v. 10.2, Boston, MA, USA).

### 2.8. Luciferase Reporter Assay

To perform the binding confirmation assay, we designed two wild-type (WT) constructs, each harboring one of the two predicted 3′UTR binding regions of murine Meis1 to miR-499-5p (WT1 or WT2) (Table 1). Firstly, each set of forward and reverse WT oligonucleotides was annealed to generate a double-stranded DNA insert. The pmirGLO-Dual Luciferase microRNA Target Expression Vector (Promega, Madison, WI, USA) was then linearized using *XbaI* and *SacI* to allow for the ligation of the annealed oligonucleotides downstream to the luciferase reporter gene. Later, Dh5α-competent bacterial cells were transformed with either WT1 or WT2 constructs, which were then isolated using a Mini Prep kit (Qiagen, Germany). In total, 10 nM of miR-499-5p mimics were co-transfected with 500 ng of the respective vector using Lipofectamine 3000 in HEK293 cells. 48 h post-transfection, the luciferase activity was measured using Hidex Sense microplate-reader and the Steady-Glo Luciferase Reporter Assay Kit (Promega, USA) following the manufacturer’s guidelines by normalizing the Firefly to the Renilla luminescence. For the binding region, which showed a decrease in luciferase activity upon miR-499-5p mimicking, we constructed, as described above, a control mutant (Mut) vector, in which the binding region nucleotides were omitted (Table 1).

### 2.9. BrdU Incorporation Assay

Proliferation of C166 cells was assessed 48 h following transfection with miR-499-5p mimics or si-Meis1 or si-Malat1, using Hidex Sense microplate-reader and the BrdU Cell Proliferation ELISA Kit (Roche Applied Biosystems, Berlin, Germany), as per the manufacturer’s protocol.

### 2.10. Bioinformatic and Statistical Analysis

Prediction of microRNAs targeting Meis1 at the 3′ untranslated region (3′UTR) was carried out using TargetScan 8.0/software: http://www.targetscan.org (accessed on 19 January 2020). The in silico tool ENCORI (https://rnasysu.com/encori/) was used to predict lncRNAs that potentially sponge the selected miRNA in both mice and humans. NCBI Protein and Nucleotide BLAST programs were used to align the human and mouse sequences of Meis1 and ncRNAs, respectively. miRbase 22.1 bioinformatic software: https://mirbase.org/ (accessed on 20 January 2020) was used to retrieve the mature miRNA sequence in human and mouse species.

For proteomics data, differentially expressed proteins (DEPs) were obtained for each treatment using a *p*-value < 0.05 and log2 fold-change ± 1.5. The biological process aspects of gene ontology (GO), KEGG, and Reactome were selected to perform functional enrichment analysis for the DEPs using the gprofiler2 (parameters: organism = *mus musculus*; significant = true; user threshold = 0.05; evcodes = true; correction method = Benjamini-Hochberg FDR; domain scope = known) R package version 4.4.2. The terms were selected based on their significance (FDR < 0.05) and their relation to cell cycle, cell division and cell proliferation.

All experiments were performed in at least two biological replicates with a minimum of three technical repeats. A normal distribution was confirmed using a normality test and a parametric unpaired student’s t-test was applied in all experiments. All bar graphs represent mean ± standard deviation (SD). *p* < 0.05 is considered statistically significant. Figures were generated using GraphPad Prism 5.00 software or R packages using ggplot2 (v3.5.1).

## 3. Results

### 3.1. miR-499-5p Acts as an Upstream Regulator of Meis1

**Figure 1:**
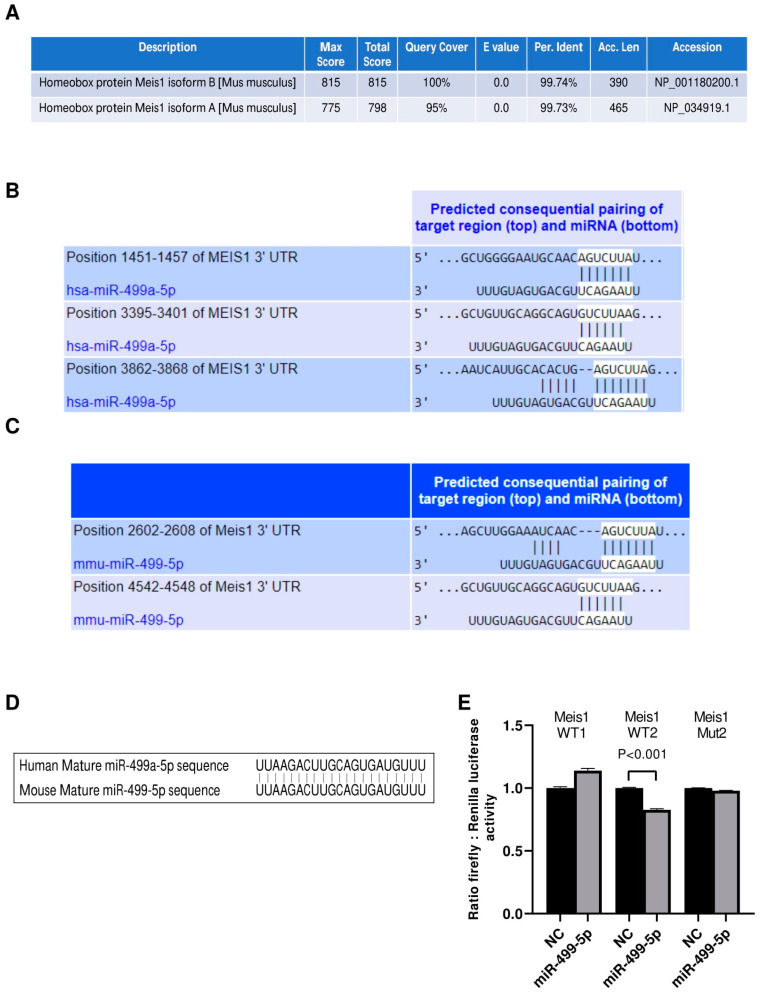
miR-499-5p is an upstream regulator of Meis1. (**A**) Amino acid sequence alignment between the human Meis1 protein (query) and the two isoforms of the mouse Meis1 protein, conducted using the NCBI Protein BLAST tool, showing over 99% identity. (**B**) Schematic illustration of the predicted binding sites between the 3′UTR of human Meis1 mRNA and the seed sequence of human miR-499a-5p, based on TargetScan software. (**C**) Schematic illustration of the predicted binding between 3’UTR of mouse Meis1 mRNA and the seed sequence of mouse miR-499-5p, based on TargetScan software. (**D**) Sequence alignment of the mature miR-499-5p in human and mouse showing 100% identity. (**E**) Luciferase reporter assay validating the targeting of Meis1 3’UTR by miR-499-5p at one of the predicted binding sites (*n* = 2). A parametric unpaired student’s *t*-test is used for statistical analysis. Data are expressed as the mean ± SD.

Initially, we investigated the conservation of our protein of interest between human and mouse species using the NCBI protein BLAST tool. Analyses revealed that Meis1 is a highly conserved protein with more than 99% identity between the human query sequence and both of the mice Meis1 protein isoforms (Figure 1A). Even though the longer mouse Meis1 isoform, isoform A, has a lower score and query coverage percentage than the shorter isoform B, both sequences remain almost identical to human Meis1.

We then proceeded to identify miRNAs that potentially target the 3′ UTR of Meis1 in both human and mouse species using the in silico analysis tool TargetScan. Our analysis revealed that hsa-miR-499a-5p and its homolog mmu-miR-499-5p are among the top scoring miRNAs predicted to bind to Meis1 (Figure 1B,C). Since miR-499a-5p has previously been shown to be associated with cell proliferation, we selected this miRNA for further analysis to determine whether it promotes its regulatory functions on cell proliferation through regulating Meis1. Notably, hsa-miR-499a-5p has three binding sites on human Meis1, whereas mmu-miR-499-5p has two. According to TargetScan, the predicted binding sites in murine Meis1 have different conserved branch lengths (CBLs) and preferentially conserved targeting (PCT) scores; for WT1, CBL= 1.854 and PCT < 0.1, whereas for WT2, CBL = 3.422 and PCT= 0.31. Remarkably, not only Meis1 is conserved amongst both species, but also the predicted targeting miRNA, miR-499-5p, is conserved, sharing identical mature miRNA sequences (Figure 1D) which might provide insights about the similar role of miR-499-5p in regulating Meis1 in humans and mice.

To confirm the binding of miR-499-5p to mouse Meis1 at the predicted binding regions, a luciferase binding assay was employed. miR-499-5p mimics were co-transfected with the different Meis1 WT pmirGLO constructs (WT1 or WT2). A significant 17% decrease in the relative luciferase activity was observed when mimicking with miR-499-5p compared to the NC, but only in the case of the Meis1 WT2 construct (Figure 1E). Moreover, the effect was no longer observed when the nucleotides in the 3′UTR seed sequence were omitted (Mut2) (Figure 1E). Luciferase activity remained unaffected by miR-499-5p mimics in cells co-transfected with the WT1 construct. This suggests direct targeting and transcriptional inhibition of Meis1 by the binding of miR-499-5p at the second predicted binding site (position 4542-4548).

### 3.2. miR-499-5p Affects Meis1 mRNA and Protein Expression

**Figure 2:**
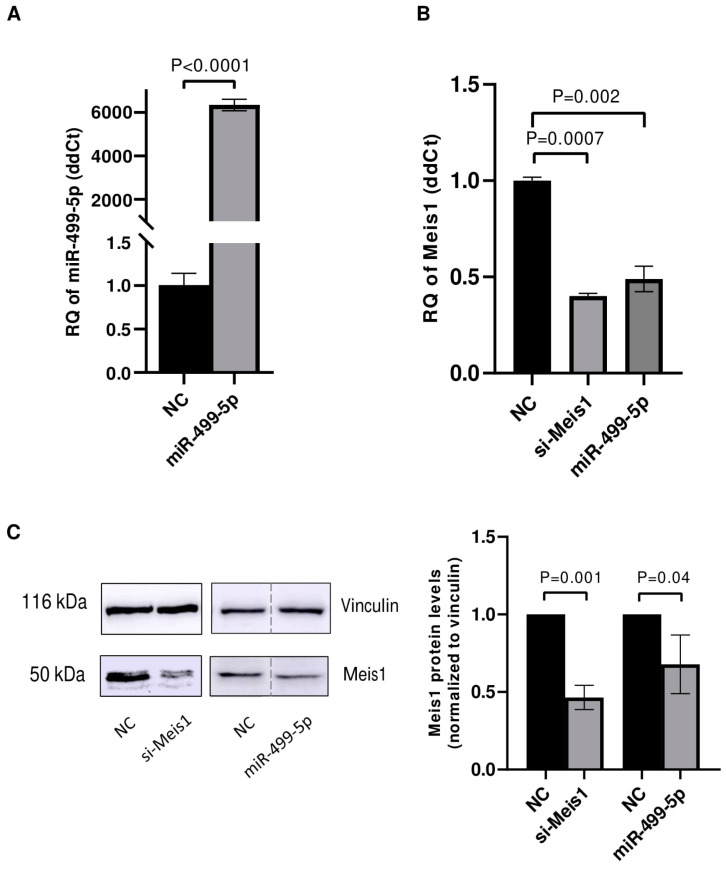
miR-499-5p affects Meis1 mRNA and protein expression. Impact of mimicking miR-499-5p in C166 cells on the levels of (**A**) miR-499-5p, reflective of the transfection efficiency and (**B**) Meis1 mRNA measured by RTqPCR (*n* = 3). (**C**) Meis1 protein assessed by Western blot and quantified using ImageQuant software (*n* = 3). The dotted line indicates spliced lanes from the same blot. A parametric unpaired student’s t-test is used for statistical analysis. Data are expressed as the mean ± SD.

After confirming the direct binding of miR-499-5p to Meis1 through one of the predicted binding sites (WT2), transfection experiments were conducted in C166 cells to assess the impact of this miRNA on Meis1 expression levels. First, the transfection efficiency of miR-499-5p was tested and showed a significant increase of more than 6000 folds in the levels of miR-499-5p (Figure 2A). Upon mimicking, Meis1 mRNA expression was downregulated by 50% compared to the NC. This reduction was similar to the 61% downregulation achieved upon Meis1 knockdown using specific siRNAs, which served as a positive control (Figure 2B).

Similarly, the impact of miR-499-5p on Meis1 protein levels was evaluated using Western blot. The band intensities appeared to be diminished upon mimicking of miR-499-5p and knockdown of Meis1 (Figure 2C). Quantification of the bands revealed a 32% reduction in the relative Meis1 protein levels after miR-499-5p mimicking compared to the NC, and a 55% reduction using si-Meis1 (Figure 2C). Taken together, miR-499-5p suppresses Meis1 at both the mRNA and protein levels.

### 3.3. Malat1 Sponges miR-499-5p to Regulate Meis1 mRNA and Protein Expression

**Figure 3:**
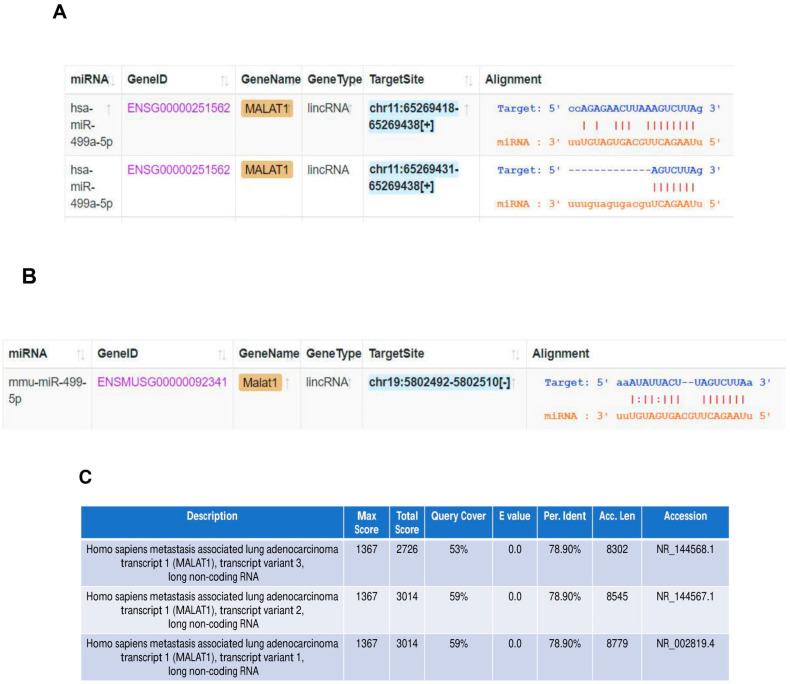
Malat1 sponges miR-499-5p. (**A**) Schematic illustration of predicted binding sites between 3’UTR of human Malat1 lncRNA and the seed sequence of human miR-499a-5p, based on Encori software. (**B**) Schematic illustration of predicted binding site between 3’UTR of mouse Malat1 lncRNA and the seed sequence of mouse miR-499-5p. (**C**) Nucleotide sequence alignment between human Malat1 lncRNA (query) and the three transcript variants of mouse Malat1 lncRNA, conducted using NCBI Nucleotide BLAST tool, showing almost 79% identity.

The in silico tool ENCORI was used to predict the sponging of miR-499-5p by Malat1 in both humans and mice (Figure 3A,B). We found that miR-499a-5p is predicted to target human MALAT1 in two binding sites (Figure 3A), while the mouse miRNA has only one predicted binding site to the mouse Malat1 (Figure 3B). In addition, BLAST nucleotide alignment between the human Malat1 sequence (query) and the three Malat1 variants in the mouse revealed a 53% to 59% query coverage with approximately 79% identity (Figure 3C). These bioinformatic predictions indicate that the human and mouse Malat1 are somewhat similar but not identical. Our computational predictions were validated in vitro by other studies, where one of them has shown that miR-499-5p is directly sponged and regulated by lncRNA Malat1 in mice neurons [35], whereas the other has more recently confirmed this targeting in a cardiac human fibroblast cell line [36]. Therefore, we sought to explore the effect of manipulating these ncRNAs on Meis1 regulation.

**Figure 4:**
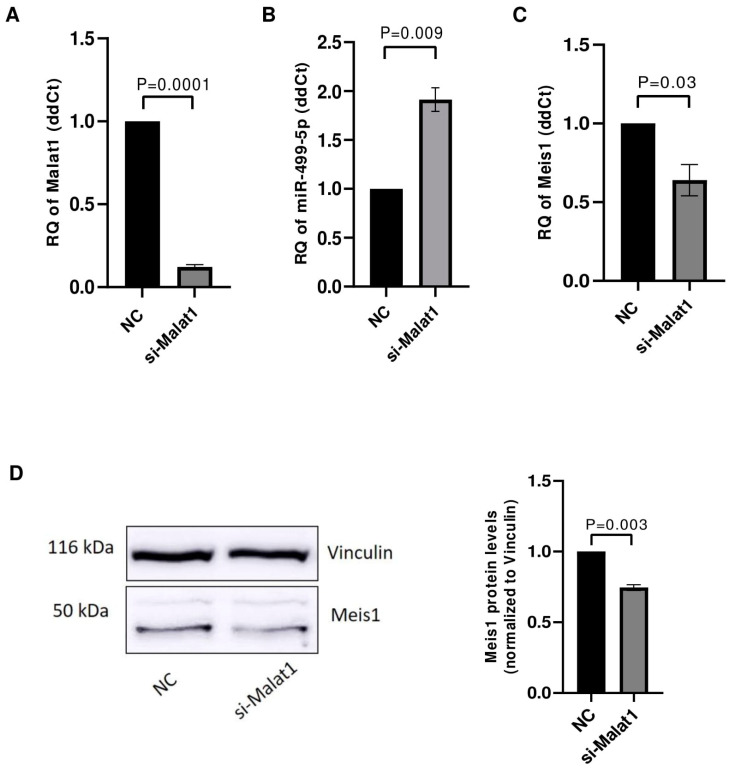
Malat1 knockdown affects miR-499-5p and Meis1 expression. (**A**) Knockdown efficiency of si-Malat1 in C166 cells (*n* = 3). (**B**) Effect of Malat1 knockdown in C166 cells on miR-499-5p (*n* = 3). (**C**) Meis1 mRNA expression quantified by RTqPCR (*n* = 3). (**D**) Meis1 protein expression assessed by Western blot and quantified using ImageQuant software (*n* = 3). A parametric unpaired student’s t-test is used for statistical analysis. Data are expressed as the mean ± SD.

The impact of Malat1 knockdown on its downstream targets miR-499-5p and Meis1 was tested in C166 cells. After ensuring the efficient knockdown of Malat1 with an 88% downregulation (Figure 4A), the levels of miR-499-5p were assessed using RTqPCR. miR-499-5p expression was significantly upregulated by approximately 2 folds (Figure 4B), validating Malat1 as an upstream regulator of this miRNA. Meis1 levels were also quantified in Malat1-knockdown cells. Similar to the previously observed effect of miR-499-5p mimicking, Malat1 knockdown caused a 36% reduction in Meis1 mRNA expression (Figure 4C). In parallel, the inhibition of Malat1 led to a reduction in Meis1 protein levels (Figure 4D), with a 26% decrease in expression (Figure 4D).

### 3.4. Impact of Malat1, miR-499-5p and Meis1 on Cell Proliferation

**Figure 5:**
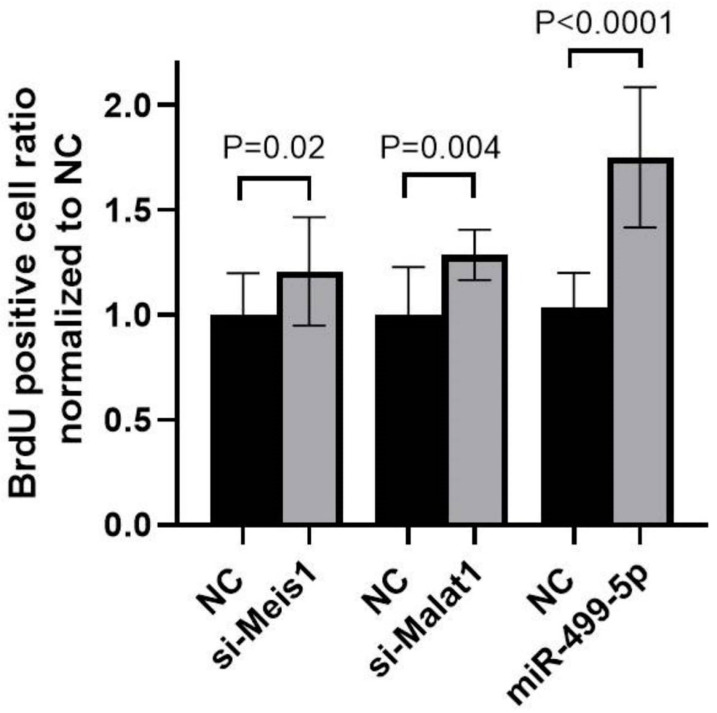
Impact of Malat1, miR-499-5p, and Meis1 on cell proliferation. Impact of Malat1 knockdown, miR-499-5p mimicking, and Meis1 knockdown on cellular proliferation in C166 cells assessed by the BrdU incorporation assay (*n* = 3). A parametric unpaired student’s t-test is used for statistical analysis. Data are expressed as the mean ± SD.

To explore the functional impact of manipulating Malat1, miR-499-5p or Meis1, a BrdU cellular proliferation assay was performed in C166 cells. A 1.3-fold increase in cell proliferation was detected after inhibiting Malat1, while mimicking miR-499-5p promoted cell proliferation by 1.7-fold compared to the NC. These increases in cell proliferation mirrored the 1.2-fold increase observed when Meis1 was knocked down using specific siRNAs (Figure 5). Overall, these results markedly elucidate that manipulating Malat1, miR-499-5p or Meis1 individually is sufficient to enhance cell proliferation.

### 3.5. Functional Enrichment Analyses Using Mass Spectrometry After Manipulation of Malat1, miR-499-5p and Meis1

**Figure 6:**
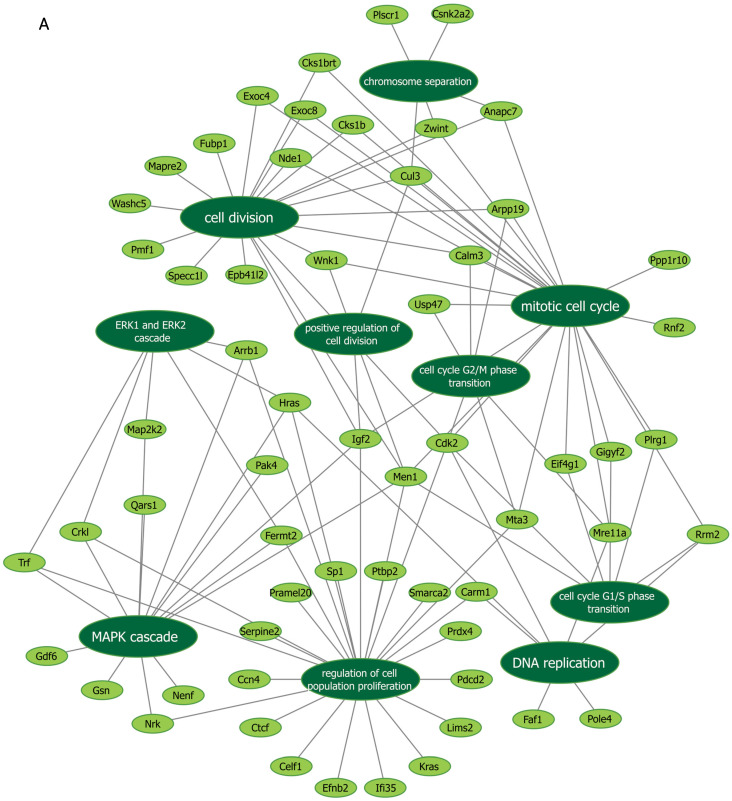

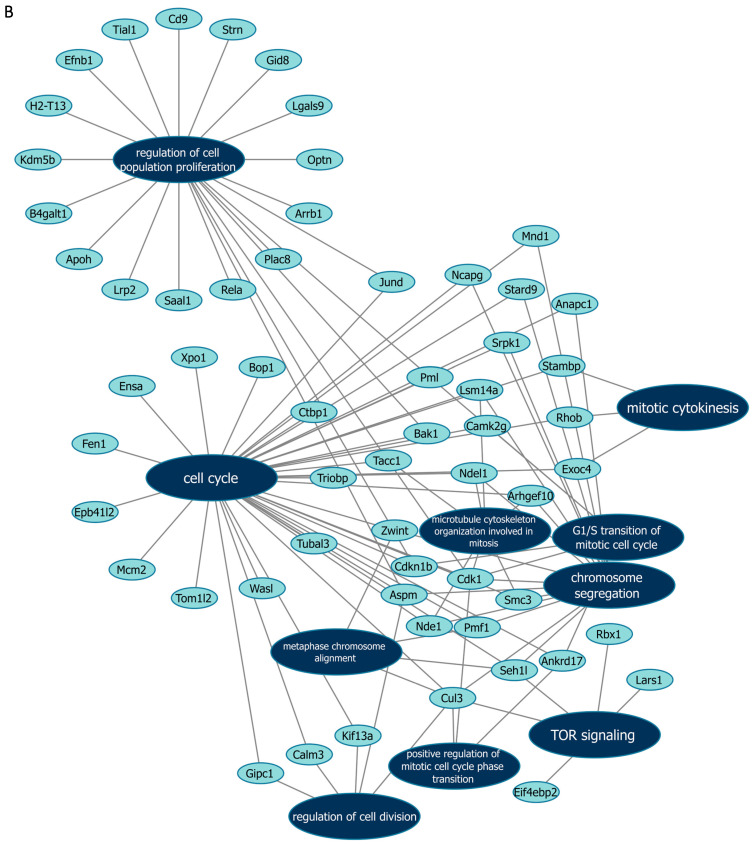

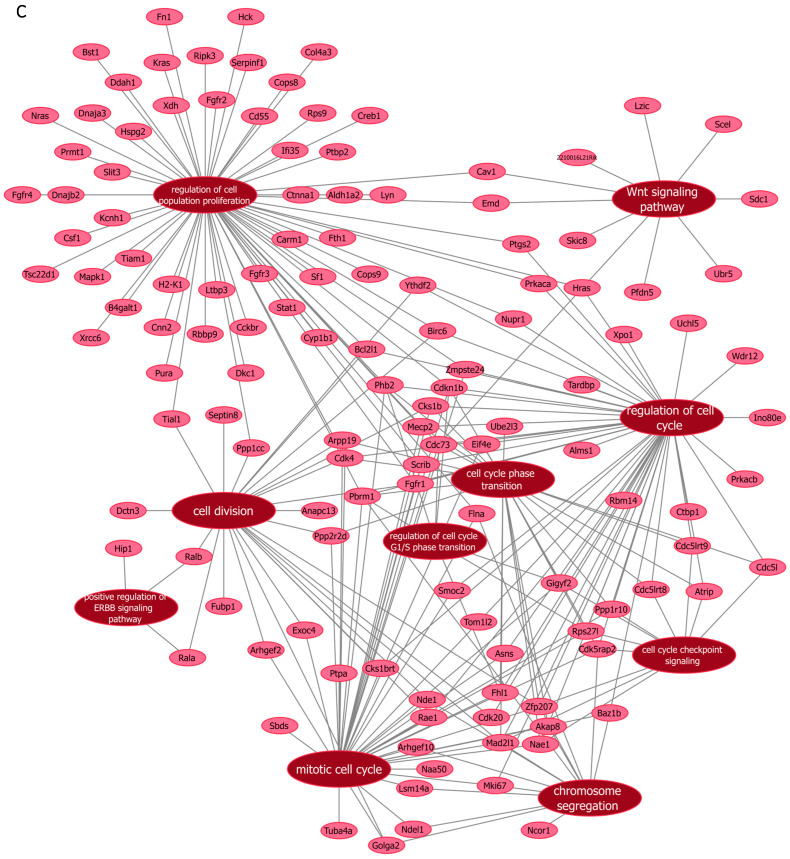
Functional networks visualizing the connections between genes and GO terms. The networks visualized in Cytoscape represent 10 GO terms and the gene symbol for DEPs within these terms, upon (**A**) Malat1 knockdown, (**B**) miR-499-5p mimicking, and (**C**) Meis1 knockdown in C166 cells.

Next, we further verified our findings on cell proliferation by performing functional enrichment analyses using mass spectrometry upon manipulation of the Malat1, miR-499-5p or Meis1. We either knocked down Malat1, forced the expression of miR-499-5p, or knocked down Meis1 in C166 cells and identified the DEPs. GO analysis revealed that the DEPs were significantly enriched in biological processes related to cell proliferation and division such as the mitotic cell cycle, regulation of cell population proliferation, chromosome separation and regulation of cell division (Figure 6 and Figure S1). Identification of key genes and proteins involved in processes such as cell division (e.g., CDK20, CKS1B, etc.) shed light on their potential interplay with Malat1, miR-499-5p and/or Meis1 (Table 2, Supplementary Data S1). Interestingly, the GO (Figure S1) as well as KEGG (Figure S2) and Reactome (Figure S3) enrichment analyses also disclosed a significant number of DEPs enriched in signaling pathways like ERBB, Wnt, MAPK, mTOR and PI3K/Akt, amongst many others. These pathways are proven to play critical roles in the regulation of cell division, cell cycle progression and proliferation [37,38,39,40,41,42,43,44]. Functional networks depicting GO gene cluster interactions allow for the visualization of DEPs in common between the different enriched GO terms (Figure 6).

Furthermore, KEGG enrichment analysis shows that the knockdown of Meis1 and Malat1 affected proteins that play a role in various cancer pathways (Figure S2A,B), which is consistent with their previously reported functions in tumorigenesis [45,46,47]. Notably, all the results from KEGG and Reactome analyses have an FDR of less than 0.05, indicating high confidence in the enriched pathways identified.

## 4. Discussion

Meis1 is a transcription factor characterized by its pleiotropic functions and diverse interacting partners. It plays pivotal roles in various biological processes including cell differentiation, development, and the cell cycle, and has also been implicated in tumor development [2,3,4,5,6,7]. It is therefore evident that regulating Meis1 would provide potential therapeutic avenues in many pathological conditions like heart failure, cancer, and developmental disorders.

ncRNAs offer a versatile tool to fine-tune the expression of specific genes. Notably, several ncRNAs have been reported to regulate Meis1 [28,29,30,31,32,33]. Here, we have identified for the first time miR-499-5p as a novel regulator of Meis1. Our bioinformatic analyses show that the human miR-499a-5p and its mouse homolog miR-499-5p, which share a 100% identical mature sequence, are predicted to target the 3’UTR of Meis1 in both humans and mice, respectively. Forcing the expression of miR-499-5p had a statistically significant impact on Meis1, downregulating its mRNA and protein expressions.

A selective binding to only one of the two predicted binding sites (WT2) was further confirmed by luciferase assay. The selective binding of miR-499-5p to WT2 might be explained by a higher CBL score as well as a higher PCT score compared to the other predicted binding site. CBL reflects the evolutionary distance over which the miRNA binding site has been conserved, while PCT estimates the likelihood of conservation for an miRNA binding site at the 3’UTR of a gene due to selective pressure, rather than by chance. Higher CBL and PCT scores indicate that this binding site has been conserved for a long period across many species, suggesting that it likely has a critical regulatory function [48].

We were then interested in identifying an lncRNA that potentially sponges miR-499-5p and subsequently regulates Meis1. Building on prior knowledge that Malat1 acts as a ceRNA sequestering miR-499-5p [35,36], we have identified this lncRNA as an indirect regulator of Meis1. Of note, Malat1 is one of the first lncRNAs to be discovered and has been implicated in the regulation of numerous ncRNAs and genes [49,50], yet this is the first time for Malat1 to be associated with Meis1. Extensively studied in diverse biological contexts and tissues across both humans and mice, Malat1 is recognized for its roles in normal cellular functions, including gene expression regulation [51,52], mRNA metabolism [53] and RNA processing [54]. It has been demonstrated that the elevated MALAT1 levels in normal tissues confer protection against osteoporosis and bone metastasis [55]. Furthermore, its involvement in pathological conditions such as cardiac hypertrophy and myocardial infarction [56,57], as well as metastasis and progression of several cancer types [45,55,58,59], is well established. Thus, harnessing Malat1 to influence the expression of other ncRNAs or genes is of great potential and could be significantly relevant in vivo in both physiological processes and pathological conditions.

Interestingly, our results show that the knockdown of Malat1 caused an increase in miR-499-5p expression, and a subsequent decrease in Meis1 at the mRNA and protein levels. These findings might suggest that Malat1 not only functions as an miR-499-5p decoy, altering its activity on its downstream targets, but can also affect its expression levels. While the knockdown of murine Malat1 had no effect on miR-499-5p levels in neurons [35], our RT-qPCR analysis is consistent with the recent finding by Chuang et al. [36], demonstrating the upregulation of miR-499a-5p upon MALAT1 knockdown. Analogous observations on miRNAs miR-372 and miR-448 were previously reported following the knockdown of their sponging lncRNAs HULC and NEAT1, respectively [60,61].

To decipher the increase in the expression of sponged miRNAs, a number of hypotheses have been proposed. Many suggest that lncRNAs may facilitate the degradation of their sponged miRNAs through target-directed miRNA degradation (TDMD) [62,63,64], a process to be distinguished from the potential ceRNA competition. In the past decade, it has become clear that the sophisticated mechanisms of interplay between miRNA and lncRNA depend on several factors. These include the expression levels or the abundance of both lncRNA and its targeted miRNA [62], which largely define their stoichiometric relationship, and their degree of complementarity [62,65].

On the one hand, the ceRNA mechanism is most likely to occur when miRNA concentrations are relatively low compared to their target. In this scenario, the limited availability of miRNAs results in the reduced suppression of their targets, as multiple targets compete for the same miRNA molecules [66]. On the contrary, a high miRNA-to-target ratio is less susceptible to competition [66,67]. A “mixed-affinity model” has been described by Denzler et al. [67], explaining that miRNAs interact with target sites that vary in binding strength, including high-affinity (strongly complementary) and low-affinity (weaker) sites. Nevertheless, high-affinity sites are more effective in sequestering miRNAs, while low-affinity sites contribute cumulatively when present in large numbers.

On the other hand, the TDMD mechanism entails the degradation of bound miRNAs. This is especially triggered by the extensive complementarity at the miRNA 3’ end, combined with a central bulge between a target RNA and the miRNA [66]. Besides miRNA degradation, TDMD involves post-transcriptional 3’ end tailing of the miRNA sequence as well as trimming and unloading from the Argonaute protein. Complementarity alone does not suffice for TDMD, as the process also depends on specific context-dependent quantitative factors, which are pronounced at high target-to-miRNA ratios. Remarkably, even targets with relatively low levels can effectively induce TDMD, given that their expression falls within a stoichiometric balance relative to their corresponding miRNAs [66]. Most of the studies suggest that ceRNA or the TDMD mechanisms have conducted in vitro manipulations; therefore, the physiological relevance and whether these processes occur naturally in vivo is debatable [67]. Overall, our findings suggest a potentially complex relation between Malat1, miR-499-5p and Meis1, which might form an axis. Given that the Meis1 expression has been shown to influence cell proliferation, as previously reported in the literature, we next explored whether the identified ncRNA regulators also affect this process. We demonstrate that manipulating each of our players individually, either by knocking down Malat1 or Meis1, or mimicking miR-499-5p, is sufficient to enhance endothelial cell proliferation.

miR-499-5p has been reported to control proliferation in cardiomyocyte progenitor cells [68]. Like its target Meis1, miR-499-5p exhibits many roles that can often appear conflicting depending on the cell type, interacting partners and context. Even though many studies support a tumor suppressive function for miR-499a-5p in different human tumors such as osteosarcoma [69], cervical cancer [70], acute myeloid leukemia [71] and glioma [72], our data reveal a pro-proliferative role possibly through modulating Meis1 mRNA and protein expression. Our findings are consistent with the results by Ouyang et al. [43], He S et al. [73] and Sheng et al. [74], showing an increase in cell proliferation in pancreatic cancer, lung adenocarcinoma, and vascular smooth muscle, respectively. As for Malat1, it is mostly recognized for promoting proliferation, migration, invasion, and angiogenesis [75,76,77,78]. Nevertheless, our BrdU assay results show that the inhibition of Malat1 induces the proliferation of C166 cells. This could point towards cell-, tissue-, or disease-specific effects for these ncRNAs.

Then, to further elucidate the biological processes and key pathways involving Malat1, miR-499-5p or Meis1, we explored the impact of manipulating each of these players through a proteomics-based approach. Interestingly, our mass spectrometry-based proteomics data further substantiated the impact of Malat1, miR-499-5p and Meis1 on cell proliferation. By revealing that the enriched DEPs are strongly associated with cell proliferation, cell division and the cell cycle, as well as their related pathways like Wnt, mTOR, Hedgehog and PI3K/Akt, our data align with the previous literature discussing the specific functions of Meis1, Malat1 and miR-499-5p. To illustrate, miR-499a-5p was previously shown to promote cell proliferation and epithelial–mesenchymal transition (EMT) via the mTOR pathway [73], while Malat1 was found to regulate EMT via PI3k/Akt and Wnt signaling pathways [79]. Moreover, this lncRNA modulates cell cycle progression and affects p53 and its target genes in fibroblasts [52]. Additionally, in acute myeloid leukemia, the activity of Meis1 has been shown to be dependent on the activation of the Wnt/β-catenin signaling pathway [80]. Notably, certain biological processes and pathways such as chromosomal segregation, cell cycle phase transition and Rap1 signaling were commonly affected by the manipulation of Malat1, miR-499-5p or Meis1. This indicates a potential interplay, further supporting evidence of an axis between the three players.

To extend the implications of our current findings, future work should focus on investigating the effects of Malat1, miR-499-5p and Meis1 on the cell cycle and in other cellular contexts, which could provide deeper insights into their intricate regulatory roles in different processes. Moreover, given that our study was conducted in vitro, a natural progression would be to extend this research to in vivo models to further validate and explore the findings in a more biologically relevant context. This would allow for a better understanding of their implications in complex physiological environments.

In conclusion, this paper underscores the novel interplay between Malat1, miR-499-5p and Meis1 as modulators of cell proliferation and cell cycle progression through impacting several downstream signaling cascades. ncRNAs can therefore be used to fine-tune Meis1 expression with potential applications in diseases in which the regulation of cell proliferation would be advantageous.

## Figures and Tables

**Table 1:** Forward (F) and reverse (R) insert sequences for wild-type (WT) and mutant (Mut) 3′ UTR constructs where the seed sequence is underlined.

Oligonucleotide Name	Sequence (5′ to 3′)
MEIS1-499 WT1 F	CAGCTTGGAAATCAACAGTCTTATTACCTCATCATGGAT
MEIS1-499 WT1 R	CTAGATCCATGATGAGGTAATAAGACTGTTGATTTCCAAGCTGAGCT
MEIS1-499 WT2 F	CGCTGTTGCAGGCAGTGTCTTAAGGAGACTGGTAGGAGT
MEIS1-499 WT2 R	CTAGACTCCTACCAGTCTCCTTAAGACACTGCCTGCAACAGCGAGCT
MEIS1-499 Mut2 F	CGCTGTTGCAGGCAGTGGAGACTGGTAGGAGT
MEIS1-499 Mut2 R	CTAGACTCCTACCAGTCTCCACTGCCTGCAACAGCGAGCT

**Table 2:** Gene symbols of proteins that are differentially expressed in significantly enriched GO terms, Malat1 knockdown, miR-499-5p mimicking and Meis1 knockdown.

Manipulation	GO Term	Gene Symbol
si-Malat1	Cell cycle G1/S phase transition	Rrm2, Cdk2, Eif4g1, Gigyf2, Men1, Plrg1
	Cell cycle G2/M phase transition	Calm3, Usp47, Mta3, Arpp19, Cdk2, Mre11a
	Cell division	Cul3, Specc1l, Calm3, Epb41l2, Exoc8, Fubp1, Igf2, Pmf1, Anapc7, Arpp19, Cks1b, Cdk2, Cks1brt, Exoc4, Men1, Nde1, Mapre2, Washc5, Wnk1, Zwint
	DNA replication	Hras, Rrm2, Faf1, Cdk2, Pole4, Carm1, Mre11a
	ERK1 and ERK2 cascade	Arrb1, Fermt2, Hras, Crkl, Map2k2, Trf
	MAPK cascade	Arrb1, Nenf, Fermt2, Qars1, Gdf6, Hras, Igf2, Crkl, Map2k2, Gsn, Men1, Nrk, Pak4, Trf
	Mitotic cell cycle	Cul3, Calm3, Exoc8, Igf2, Usp47, Mta3, Rrm2, Rnf2, Anapc7, Arpp19, Cks1b, Cdk2, Cks1brt, Eif4g1, Exoc4, Gigyf2, Men1, Nde1, Mre11a, Plrg1, Ppp1r10, Wnk1, Zwint
	Positive regulation of cell division	Cul3, Igf2, Men1, Wnk1
	Regulation of cell population proliferation	Arrb1, Serpine2, Celf1, Efnb2, Fermt2, Hras, Kras, Igf2, Mta3, Pdcd2, Smarca2, Sp1, Ccn4, Crkl, Cdk2, Carm1, Ifi35, Men1, Lims2, Nrk, Prdx4, Ptbp2, Pramel20, Ctcf, Trf
	Chromosome separation	Cul3, Anapc7, Csnk2a2, Plscr1, Zwint
miR-499-5p	G1/S transition of mitotic cell cycle	Ankrd17, Camk2g, Cdk1, Cdkn1b, Pml
	Chromosome segregation	Anapc1, Cdk1, Smc3, Lsm14a, Pmf1, Arhgef10, Aspm, Mnd1, Ndel1, Nde1, Zwint, Cul3, Ncapg, Srpk1, Seh1l, Stard9
	Cell cycle	Ankrd17, Ctbp1, Ensa, Fen1, Camk2g, Anapc1, Tacc1, Mcm2, Cdk1, Jund, Cdkn1b, Smc3, Lsm14a, Pmf1, Bak1, Arhgef10, Aspm, Mnd1, Ndel1, Nde1, Tubal3, Pml, Zwint, Xpo1, Calm3, Rhob, Exoc4, Gipc1, Cul3, Tom1l2, Wasl, Triobp, Ncapg, Bop1, Stambp, Srpk1, Epb41l2, Seh1l, Stard9, Kif13a
	Mitotic cytokinesis	Rhob, Exoc4, Stambp
	Microtubule cytoskeleton organization involved in mitosis	Tacc1, Cdk1, Smc3, Lsm14a, Arhgef10, Ndel1, Nde1
	Positive regulation of mitotic cell cycle phase transition	Ankrd17, Cdk1, Cul3
	Metaphase chromosome alignment	Pmf1, Zwint, Cul3, Seh1l
	Regulation of cell division	Aspm, Calm3, Gipc1, Cul3, Kif13a
	Regulation of cell population proliferation	Rela, Plac8, Cdk1, Jund, Gid8, Cdkn1b, Tial1, Bak1, Kdm5b, Aspm, Lrp2, Pml, Arrb1, Optn, Lgals9, Strn, Cd9, Efnb1, H2-T13, B4galt1, Apoh, Saal1
	TOR signaling	Rbx1, Eif4ebp2, Cul3, Lars1, Seh1l
si-Meis1	Cell cycle checkpoint signaling	Cdk5rap2, Atrip, Zfp207, Rps27l, Mad2l1, Cdc5l, Cdc5lrt9, Cdc5lrt8, Ppp1r10, Nae1, Gigyf2
	Cell cycle phase transition	Cdk5rap2, Phb2, Atrip, Zfp207, Rps27l, Pbrm1, Mad2l1, Eif4e, Cks1b, Arpp19, Fhl1, Cdc73, Cdc5l, Ube2l3, Cdc5lrt9, Cdc5lrt8, Ppp2r2d, Ppp1r10, Nae1, Mecp2, Gigyf2, Cdkn1b, Cdk4, Akap8
	Cell division	Fgfr1, Cdk20, Arhgef2, Fubp1, Zfp207, Ythdf2, Nde1, Mad2l1, Exoc4, Cks1brt, Cks1b, Birc6, Arpp19, Dctn3, Anapc13, Golga2, Ppp1cc, Tial1, Septin8, Ralb, Rala, Rae1, Ppp2r2d, Cdk4, Bcl2l1
	Chromosome segregation	Cdk5rap2, Zfp207, Pbrm1, Ndel1, Nde1, Ncor1, Mad2l1, Baz1b, Arhgef10, Golga2, Flna, Mki67, Lsm14a, Akap8
	Mitotic cell cycle	Ptpa, Fgfr1, Arhgef2, Cdk5rap2, Scrib, Tuba4a, Phb2, Asns, Zmpste24, Zfp207, Tom1l2, Smoc2, Sbds, Rps27l, Pbrm1, Ndel1, Nde1, Naa50, Mad2l1, Exoc4, Eif4e, Cks1brt, Cks1b, Baz1b, Arpp19, Arhgef10, Fhl1, Golga2, Flna, Cdc73, Rae1, Ppp2r2d, Ppp1r10, Nae1, Mki67, Mecp2, Lsm14a, Gigyf2, Cdkn1b, Cdk4, Akap8
	Positive regulation of ERBB signaling pathway	Ralb, Rala, Hip1
	Regulation of cell cycle	Prkacb, Prkaca, Hras, Fgfr1, Cdk20, Cdk5rap2, Scrib, Phb2, Atrip, Asns, Zmpste24, Zfp207, Ythdf2, Wdr12, Uchl5, Tom1l2, Smoc2, Rps27l, Ptgs2, Pbrm1, Nupr1, Mad2l1, Ino80e, Eif4e, Cks1brt, Cks1b, Birc6, Baz1b, Alms1, Rbm14, Ctbp1, Fhl1, Cdc73, Cdc5l, Cdc5lrt9, Cdc5lrt8, Xpo1, Tardbp, Rae1, Ppp1r10, Nae1, Mki67, Mecp2, Gigyf2, Cdkn1b, Cdk4, Bcl2l1
	Regulation of cell cycle G1/S phase transition	Phb2, Rps27l, Pbrm1, Fhl1, Cdc73, Gigyf2, Cdkn1b
	Regulation of cell population proliferation	Slit3, Cnn2, Ptbp2, Prkaca, Hras, Mapk1, Lyn, Hck, Fgfr4, Fgfr3, Fgfr2, Fgfr1, Rbbp9, Rps9, Kras, Scrib, Pura, Phb2, Fn1, Tsc22d1, Zmpste24, Xdh, Tiam1, Stat1, Serpinf1, Ptgs2, Prmt1, Pbrm1, Nupr1, Nras, Ltbp3, Ifi35, Hspg2, H2-K1, Fth1, Emd, Dnaja3, Csf1, Cops9, Col4a3, Cav1, Carm1, Birc6, Kcnh1, Cckbr, Cops8, Ddah1, Flna, Dkc1, Cdc73, Bst1, Xrcc6, Ctnna1, Ppp1cc, Tial1, Sf1, Ripk3, Mecp2, Dnajb2, Cyp1b1, Creb1, Cdkn1b, Cdk4, Cd55, Bcl2l1, B4galt1, Aldh1a2
	Wnt signaling pathway	2210016L21Rik, Skic8, Pfdn5, Emd, Cav1, Ubr5, Cdc73, Sdc1, Scel, Lzic

## Data Availability

The original contributions presented in this study are included in the Supplementary Material. The MS proteomics data were deposited to the ProteomeXchange Consortium through the PRIDE repository with identifier PXD054558.

## Supplementary Materials

The following supporting information can be downloaded at https://www.mdpi.com/article/10.3390/cells14020125/s1. Supplementary Figure S1: Gene ontology enrichment analysis following (**A**) Malat1 knockdown, (**B**) miR-499-5p mimicking and (**C**) Meis1 knockdown; Supplementary Figure S2: KEGG enrichment analysis following (**A**) Malat1 knockdown, (**B**) miR-499-5p mimicking and (**C**) Meis1 knockdown; Supplementary Figure S3: REACTOME enrichment analysis following (**A**) Malat1 knockdown, (**B**) miR-499-5p mimicking and (**C**) Meis1 knockdown. The original contributions presented in this study are included in the supplementary material: Supplementary Figure S4: Western blot for Meis1 after miR-499-5p mimicking; Supplementary Figure S5: Western blot for Meis1 after Malat1 knockdown; Supplementary Figure S6: Western blot for Meis1 after Meis1 knockdown. The following supporting information can be downloaded: Table S1: RTqPCR primers; Supplementary Data S1: Genes encoding the differentially expressed proteins. The MS proteomics data were deposited to the ProteomeXchange Consortium through the PRIDE repository with identifier PXD054558.
