# Supplementary material for: Interaction Between Malat1 and miR-499-5p Regulates Meis1 Expression and Function with a Net Impact on Cell Proliferation
Source: Cells. 2025 Jan 16;14(2):125. doi: 10.3390/cells14020125 (PMC11764005; doi:10.3390/cells14020125)
Supplement: Supplementary file 1 [file cells-14-00125-s001.zip › Supplementary Table S1 and Figures S1 to S3.pdf]

Supplementary Table S1

Supplementary table S1: RTqPCR primers used (5′ to 3′)

Meis1 Fwd	GATGGCGCAAAGGTACGAC
Meis1 Rev	GGTACTGATGCGAGTGCAGA
hsa-mir-499a-5p	GTTAAGACTTGCAGTGATGTTTAAAA
Universal Reverse primer for miRNAs GT19	GAATCGAGCACCAGTTACG
RNU6B	ACGCAAATTCGTGAAGCGTT
Malat1 Fwd	GGGGGAATGGGGGCAAAATA
Malat1 Rev	AACTACCAGCAATTCCGCCA
SRSF4 Fwd	TGCAGCTGGCAAGACCTAAA
SRSF4 Rev	TTTTTGCGTCCCTTGTGAGC

Supplementary Figure S1

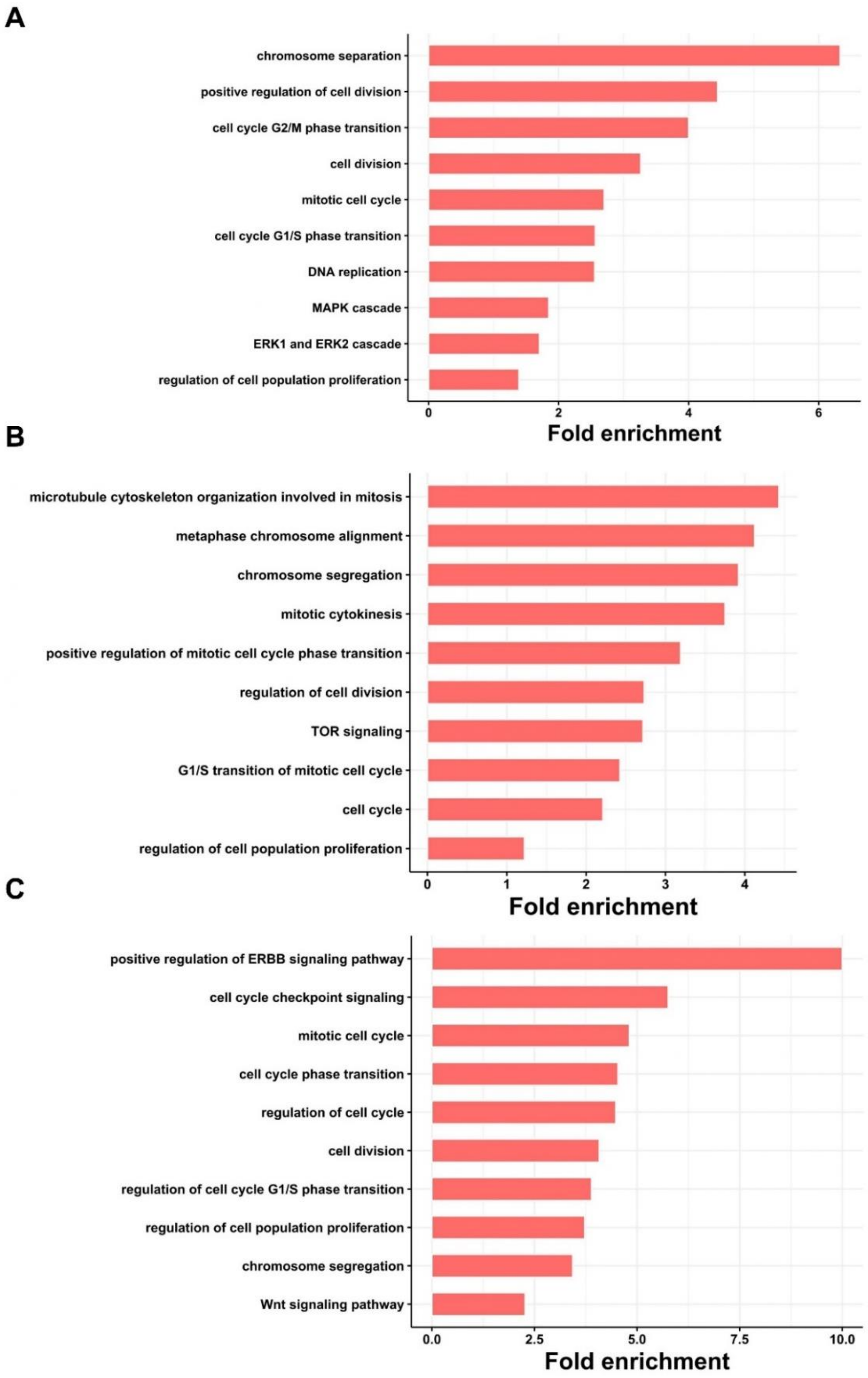

Figure S1. Gene Ontology functional enrichment analysis. Gene Ontology (GO) functional enrichment analysis of differentially expressed proteins involved in different biological processes (BP) upon **(A)** knockdown of Malat1 (n=3), **(B)** mimicking of miR-499-5p (n=3), and **(C)** knockdown of Meis1 (n=3) in C166 cells. All terms were significantly enriched, *P < 0.05.

Supplementary Figure S2

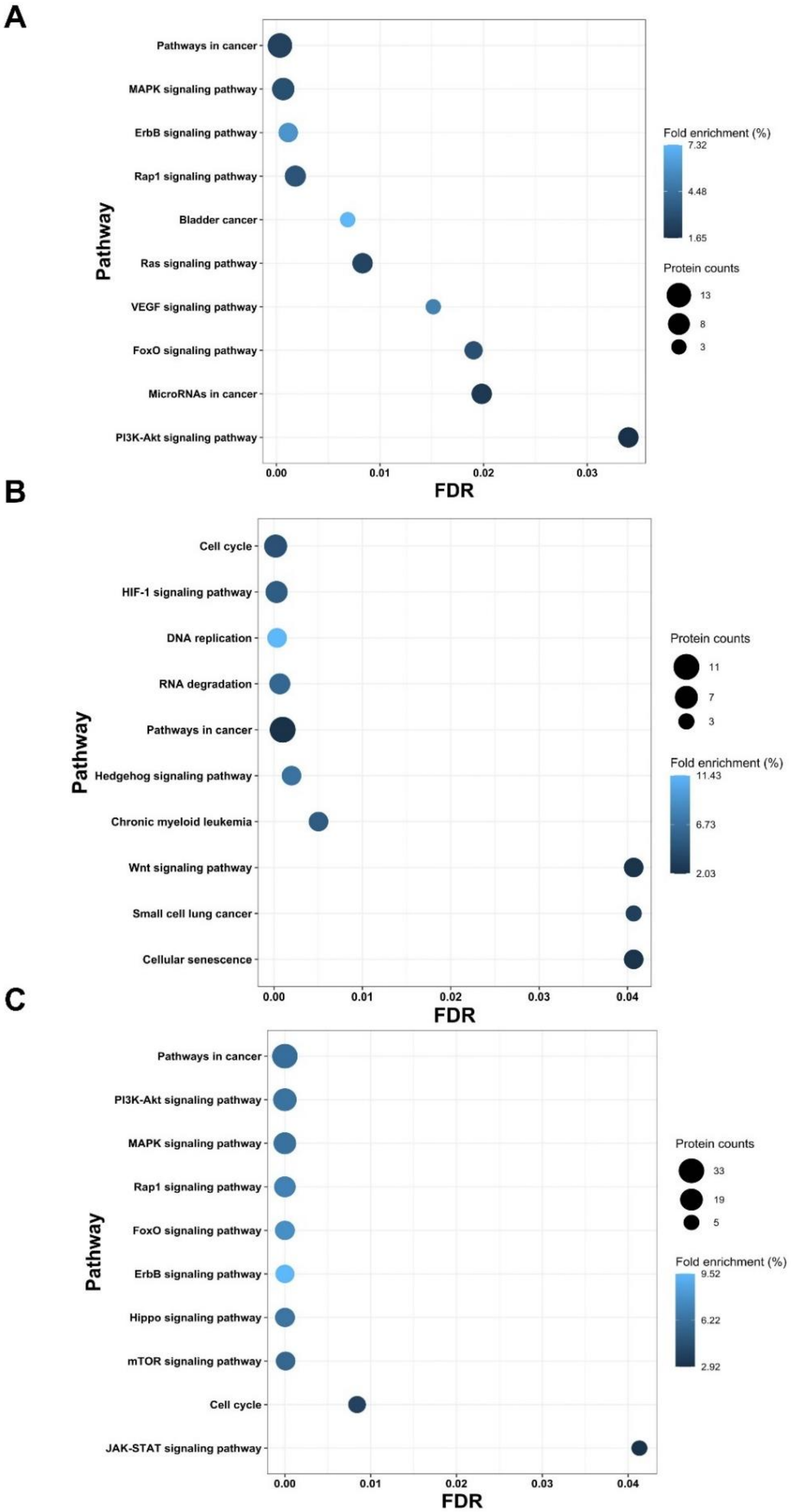

Figure S2. KEGG enrichment analysis. KEGG enrichment analysis of differentially expressed proteins involved in various pathways upon **(A)** knockdown of Malat1 (n=3)**, (B)** mimicking of miR-499-5p (n=3), and **(C)** knockdown of Meis1 (n=3) in C166 cells.

Supplementary Figure S3

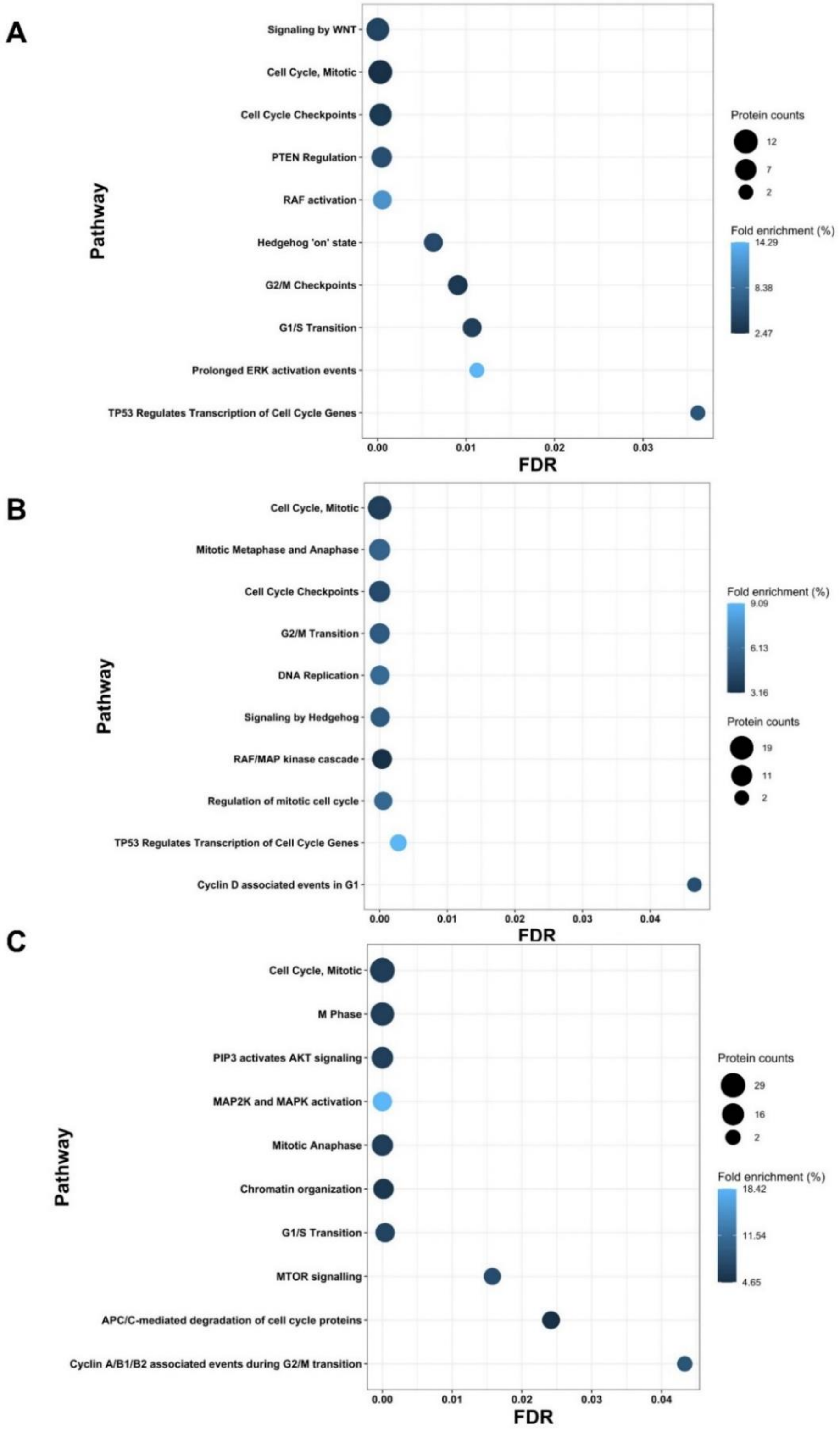

Figure 8. Reactome enrichment analysis. Reactome enrichment analysis of differentially expressed proteins involved in various pathways upon **(A)** knockdown of Malat1 (n=3), **(B)** mimicking of miR-499-5p (n=3), and **(C)** knockdown of Meis1 (n=3) in C166 cells.
