# Supplementary figures and images for: Interaction Between Malat1 and miR-499-5p Regulates Meis1 Expression and Function with a Net Impact on Cell Proliferation
Source: Cells. 2025 Jan 16;14(2):125. doi: 10.3390/cells14020125 (PMC11764005; doi:10.3390/cells14020125)

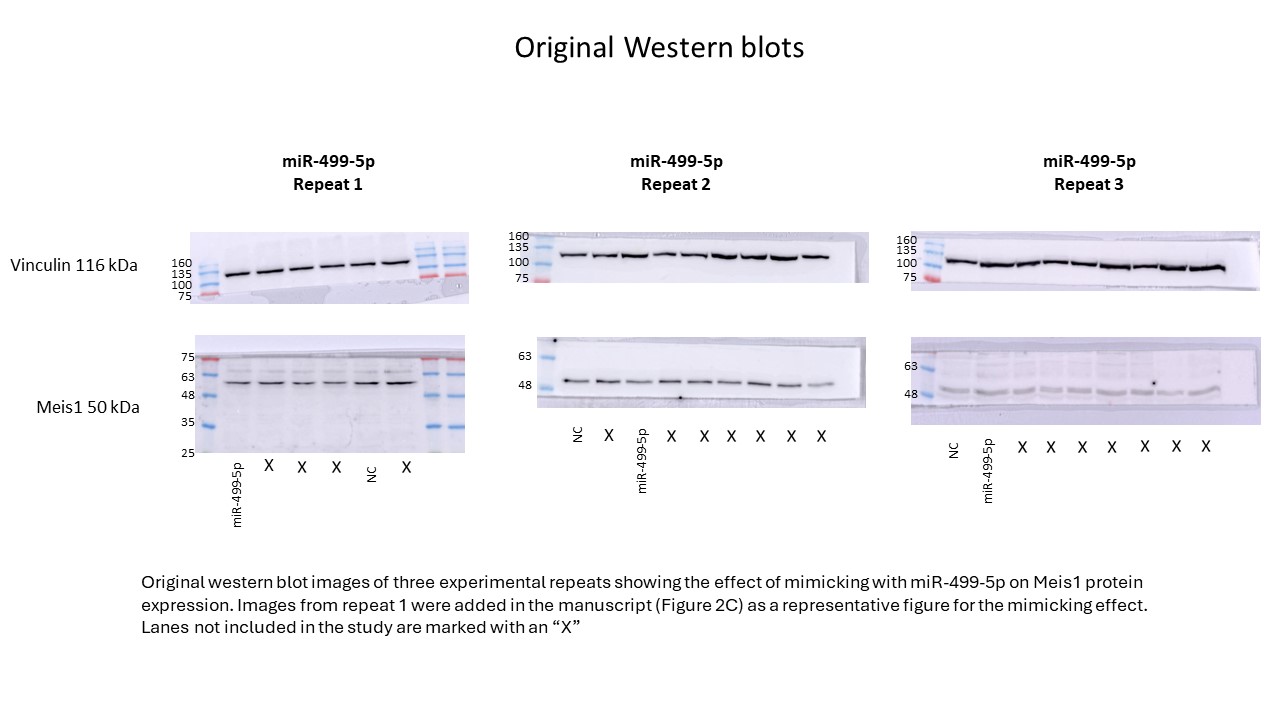

Supplement: Supplementary file 1 [file cells-14-00125-s001.zip › Supplementary Figure S4- Original Western blot for Meis1 after miR-499-5p mimicking.JPG]

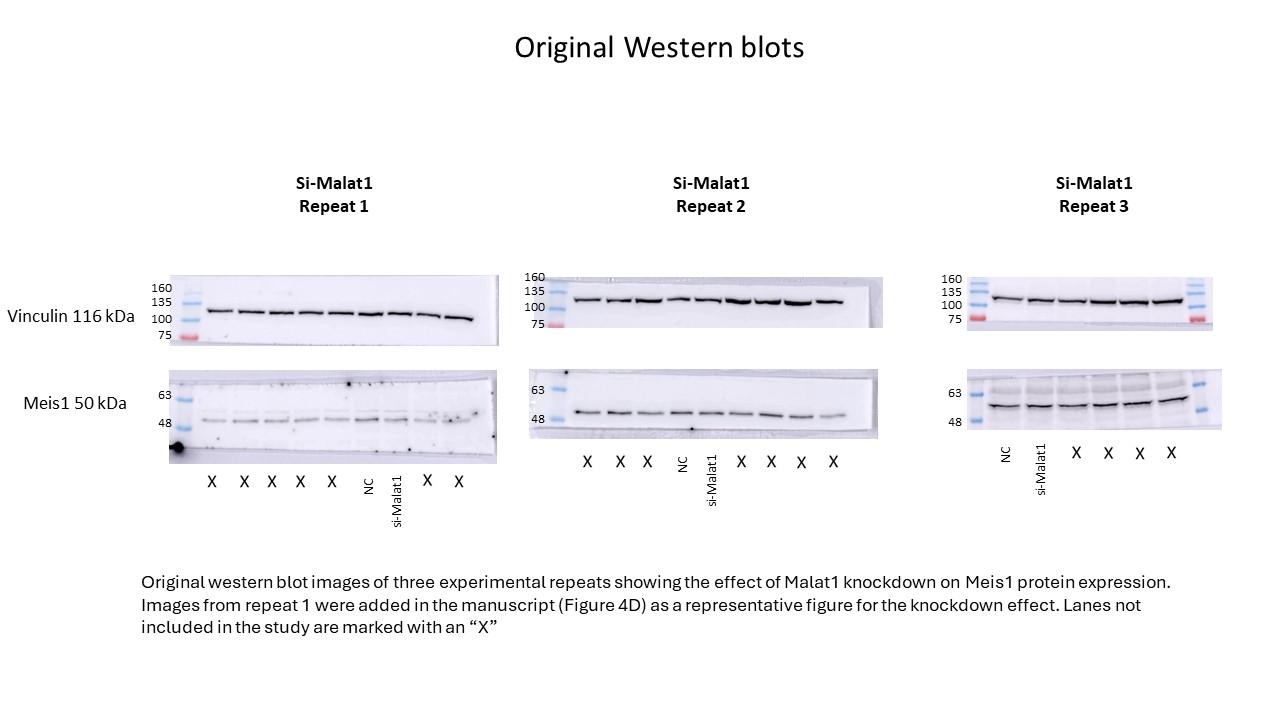

Supplement: Supplementary file 1 [file cells-14-00125-s001.zip › Supplementary Figure S5- Original Western blot for Meis1 after Malat1 knockdown.JPG]

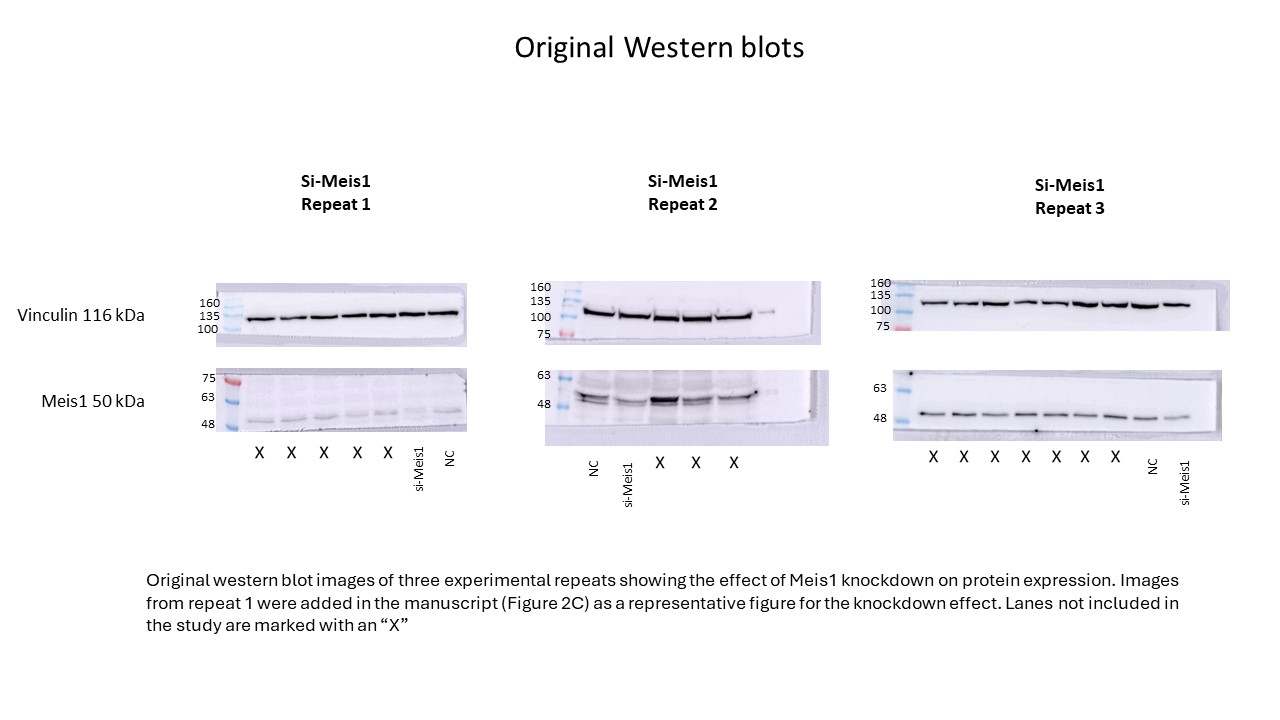

Supplement: Supplementary file 1 [file cells-14-00125-s001.zip › Supplementary Figure S6- Original Western blot for Meis1 after Meis1 knockdown.JPG]
